# Policy lessons from health taxes: a systematic review of empirical studies

**DOI:** 10.1186/s12889-017-4497-z

**Published:** 2017-06-19

**Authors:** Alexandra Wright, Katherine E. Smith, Mark Hellowell

**Affiliations:** Global Public Health Unit, Social Policy, School of Social & Political Science, University of Edinburgh, Chrystal Macmillan Building, 15a George Square, Edinburgh, EH8 9LD UK

**Keywords:** Sin taxes, Public health, Hypothecation/earmarking, Sugar tax, Fat tax, Soda tax, Tobacco, Alcohol, Systematic review

## Abstract

**Background:**

Taxes on alcohol and tobacco have long been an important means of raising revenues for public spending in many countries but there is increasing interest in using taxes on these, and other unhealthy products, to achieve public health goals. We present a systematic review of the research on health taxes, and aim to generate insights into how such taxes can: (i) reduce consumption of targeted products and related harms; (ii) generate revenues for health objectives and distribute the tax burden across income groups in an efficient and equitable manner; and (iii) be made politically sustainable.

**Methods:**

Six scientific and four grey-literature databases were searched for empirical studies of ‘health taxes’ – defined as those intended to increase the costs of manufacturing, distributing, retailing and/or consuming health-damaging products. Since reviews already exist of the evidence relating to traditional alcohol and tobacco excise taxes, we focus on other taxes such as taxes on retailers and manufacturers of unhealthy products, and consumer taxes targeting unhealthy foods, such as sugar-sweetened beverages.

**Results:**

Ninety-one peer-reviewed and 11 grey-literature studies met our inclusion criteria. The review highlights a recent, rapid rise in research in this area, most of which focuses on high-income countries and on taxes on food products or nutrients. Findings demonstrate that high tax rates on sugar-sweetened beverages are likely to have a positive impact on health behaviours and outcomes, and, while taxes on products reduce demand, they add to fiscal revenues. Common concerns about health taxes are also discussed.

**Conclusions:**

If the primary policy goal of a health tax is to reduce consumption of unhealthy products, then evidence supports the implementation of taxes that increase the price of products by 20% or more. However, where taxes are effective in changing health behaviours, the predictability of the revenue stream is reduced. Hence, policy actors need to be clear about the primary goal of any health tax and frame the tax accordingly – not doing so leaves taxes vulnerable to hostile lobbying. Conversely, earmarking health taxes for health spending tends to increase public support so long as policymakers follow through on specified spending commitments.

**Systematic review registration number:**

CRD42016048603

**Electronic supplementary material:**

The online version of this article (doi:10.1186/s12889-017-4497-z) contains supplementary material, which is available to authorized users.

## Background

Taxes directed at unhealthy products, such as alcohol, tobacco, certain foods and non-alcoholic beverages (for example ‘sugar-sweetened beverages’ - ‘SSBs’), are widely used. Historically, the primary objective of such measures has been the fiscal revenues they generate. However, as evidence of the social, economic and health harms associated with such products has accumulated, there has been increasing policy and research interest in the ability of such taxes to raise the cost of manufacturing, distributing, retailing and/or consuming unhealthy products, and thereby reducing their consumption. In particular, governments in several countries have employed taxes on tobacco and alcohol products to promote reduced consumption [1]. An international review of pricing policies and tobacco control in Europe identified extensive evidence regarding the effects of traditional taxes on tobacco products (customs duties, excise taxes and value added taxes), concluding that such taxes represent one of the most effective means of tobacco control [2]. There is also a vast amount of literature examining the relationships between product price, alcohol consumption, and alcohol-related harms. In 2009, for example, Wagenaar and colleagues published a meta-analysis of 112 studies to examine the effects of alcohol price on consumption levels. Again, the authors found a significant inverse relationship between alcohol taxes or prices and the consumption of alcohol products; a relationship which held for both light and heavy drinking patterns [3].

More recently, a number of countries have introduced new or higher taxes on a broader array of unhealthy products, or have structured taxes in new ways with the aim of increasing the cost of manufacturing, distributing, retailing and/or consuming such products. For example, since 2010, countries including Denmark, Hungary, Finland, France, Mexico and the United Kingdom have introduced sales taxes on foods or beverages deemed unhealthy; while in Scotland, a ‘public health supplement’ was introduced from 2012 to 2015 on large retailers (in effect large supermarkets) selling both alcohol and tobacco [4]. In some of these cases, which are also discussed in more detail later in this review, the revenues generated by the tax have been earmarked for specified health-related spending. Earmarking dedicates specific revenue to specific purposes, and is sometimes labelled ‘hypothecation’. Although, as we demonstrate, the literature concerning health taxes currently focuses on high income country settings, these experiences may be particularly relevant for low- and middle-income countries, in which strategies to provide universal health coverage are, it is increasingly recognized, dependent on the effective expansion of public sector financial resources [5].

While the use of alcohol and tobacco duties in changing health behaviours is well-established, we have found no publications that synthesize the empirical research on this more recent, broader range of country-specific ‘health taxes’, as mentioned in the paragraph above. This paper presents a systematic review of this research with the aim of providing insights into how such taxes can be designed to: (i) reduce consumption of targeted products and related health harms; (ii) generate revenues (especially for health-related purposes, in the case of *earmarked* taxes) and distribute the tax burden across income groups in an efficient and equitable manner and (iii) be sustained over time in the context of political constraints.

We begin with an outline of methods and then present the findings of the review. In the discussion, we consider the research gaps to be addressed and outline the lessons for future policymaking in this key area.

## Methods

We conducted a systematic search for empirical literature concerning taxes that are intended to increase the costs of manufacturing, distributing, retailing and/or consuming health-damaging products, excluding those that have already been the subject of systematic reviews (e.g. customs duties, sales taxes and VAT on alcohol and tobacco). We specifically considered the impacts of taxes in relation to the aim of this paper, stated above.

Our aim was to produce a systematic review of evidence relating to non-traditional health taxes that would be of use to policy audiences considering advocating for, or developing, new (or higher) health taxes (e.g. civil servants, politicians and health-focused non-governmental organisations [NGOs]). Our approach was informed by a study of how policy actors perceive and use health-focused systematic reviews (compared to other potential ‘evidence tools’ such as health impact assessments and cost-benefit analyses) [6]. This study found that policy actors (for example, national or local policymakers, advocates and policy campaigners, and knowledge brokers) were often frustrated by the narrow focus of systematic reviews, concerned by the number of studies excluded for quality purposes and the lack of contextual information, and disappointed by the dearth of clear policy-relevant recommendations [6]. In response, this paper provides a broad overview of what empirical studies have found about the impacts of ‘health taxes’. Given the concern raised by policy actors about the exclusion of potentially useful studies, we did not exclude studies on the basis of their quality, though we do comment on quality issues where relevant. The results are organized according to likely policy questions about health taxes, and the concluding discussion summarizes the key policy ‘lessons’ and identifies gaps and limitations in the evidence-base.

The search string for this review was developed iteratively and finalized collaboratively by the authors. The baseline search string for peer reviewed journal articles, which was developed for the PubMed database, was as follows (* indicates a truncation of the word to include all forms of that word):

(((health) AND (tobacco OR cigar* OR alcohol OR drink* OR beer OR wine OR spirits OR made-wine OR cider OR perry OR food OR soda OR beverage* OR sugar OR fat OR "sin tax")) AND (tax*[Title/Abstract] OR levy[Title/Abstract] OR levied[Title/Abstract] OR excis*[Title/Abstract])) NOT ("taxonomy" OR "syntax" OR "excision" OR "taxonomic" OR "taxonomically" OR "taxane" OR "taxi" OR "taxonic" OR parasit* OR microbial OR phenotyp*)

Databases for this review were selected after consultation with a qualified librarian on the basis of their scope and relevance. We ultimately included the following databases and aggregator sites: PubMed, OVID, Web of Science, EBSCOhost (including Academic Search Complete Business Source Complete, SocINDEX with Full Text, EconLit, and Medline), Scopus, and ProQuest (including IBSS Online and ASSIA). The baseline search string was refined for each database, and each individual search string can be found in Additional file 1. The first search was conducted in September 2015 with timeline 1990-2015. An updated search was conducted in May 2016, with timeline September 2015-May 2016. At this time, we also conducted grey literature searches in Google, the WHO website, and four grey literature databases (NBER, Global Health, Open Grey, and HISA), for the period 2000-2016.

We obtained all citations and reviewed the abstracts and full texts for relevance. Articles were included if they: (1) reported empirical data on the design, implementation, or impacts of health taxes that target unhealthy products (other than traditional tobacco and alcohol excise, already well-reviewed, or import/export duties, for reasons of feasibility); or (2) reported on empirical data (including data generated via modelling, e.g. of the likely responses of affected stakeholders to health taxes).

Studies were excluded from this review if they focused on: (1) behaviour changes caused by proportional taxes on the sale, or production for sale, of health damaging products that have already been the focus of systematic reviews (i.e. studies of consumer taxes on tobacco and alcohol products); (2) import/export duties applied to particular products where these did not have any clear health-related content or rationale; (3) quantifying the costs relating to any particular products/behaviours (for consideration for tax purposes) but not actually assessing health taxes; or (4) combined or linked interventions in which taxation was implemented alongside other kinds of intervention (and could not be separated for analysis). We also excluded publications that are not based on empirical data; (e.g. opinion pieces) and those not written in English (since no other languages were available to the research team). Publications focusing on import/export duties were excluded because they are strongly influenced by macro factors in the political economy (e.g. international trade agreements), making it difficult to ascertain their link to national public health concerns - our focus remains on taxation decisions by national governments to improve public health.

A data extraction matrix was developed in Microsoft Excel and utilized to compile the review data. The authors jointly undertook article screening and data extraction, and any uncertainties were discussed by the research team collectively. The reference lists of each article were examined for snowballing purposes which, as summarized below, led to the identification of five additional studies.

With a policy focus in mind, our approach to synthesizing the large and diverse literature was informed by the following five key questions, which our background research (initial literature review and conversations with relevant policy actors) suggested are of interest to policy audiences considering new (or additional) health taxes:

1. How (if at all) do particular health taxes change consumption behaviours and what do we know about the health-related impacts of such taxes?
2. Can health taxes on manufacturers and retailers change behaviours?
3. Do taxes that target health-damaging products succeed in providing additional fiscal revenue?
4. What is the degree of support among public and policy communities for non-traditional health taxes and are there means of increasing support?
5. What are the key critiques of health taxes and their implementation and what options exist to manage these challenges?

## Results

### Bibliographic results of literature search

**Fig. 1:**
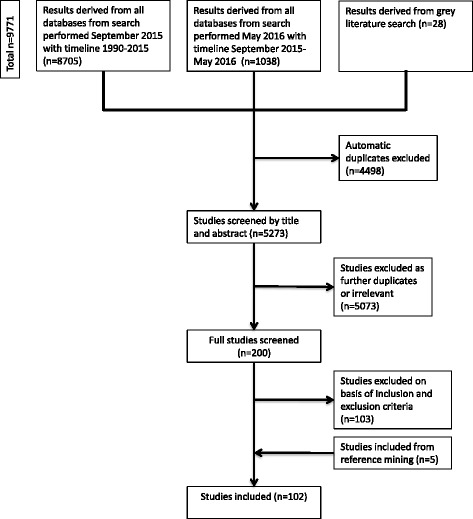
Process for identifying empirical literature on innovative health taxes

We identified 102 relevant studies (91 peer-reviewed journal articles and 11 non peer-reviewed publications), as summarized in Fig. 1.

**Fig. 2:**
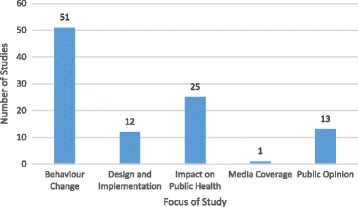
Research on innovative health taxes by study focus

As Fig. 2 summarizes, included studies largely focused on the impacts of health taxes on behavioural change, or on public health (including, in one case, the social determinants of health), with a smaller number of studies considering public opinion and issues relating to tax design and implementation, and media coverage.

**Fig. 3:**
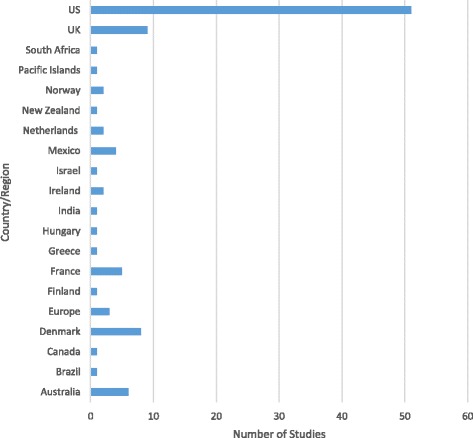
Research on innovative health taxes by geographical focus

The studies we identified focused on a range of high-income countries, and a smaller number of middle-income countries. The literature is dominated by studies of health taxes implemented in the US (51 studies) (see Fig. 3) and Europe (34 studies, either focusing on the European region as a whole or individual European countries), though this spread inevitably reflects our exclusion of non-English language articles.

**Fig. 4:**
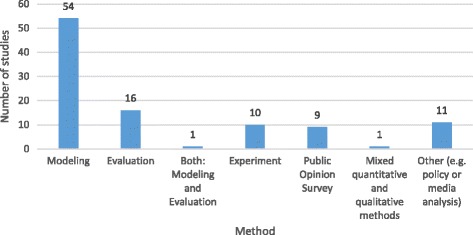
Research Methods Utilized by Included Studies

As Fig. 4 summarizes, the empirical research methods utilized in the included articles most commonly involved modelling (*n* = 54), although we also identified evaluation studies (*n* = 16), experiments (*n* = 10), public opinion surveys (*n* = 9), and alternative qualitative approaches (e.g. interviews, media analyses, citizen’s juries) (*n* = 11). We also identified two studies that employed mixed methods: one mixed modelling with evaluation and the other employed a mixed quantitative-qualitative approach.

**Fig. 5:**
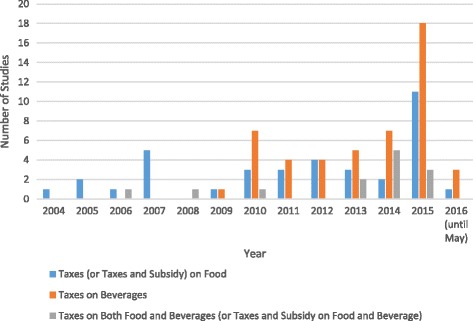
Publication year and type of taxation focus for included studies

The majority of included studies focus on taxes on food or beverage products. Figure 5 shows the number of included studies published in each year, with respect to the category of product targeted (note that, where an article focused on both food and beverages it was included in both categories, and hence the number of publications in Fig. 5 exceeds the number of included studies). This demonstrates that interest in this area seems to be increasing, with a particularly marked increase in studies of beverage taxes from 2010 onwards.

The majority of modeling studies estimated price elasticities based upon empirical data drawn from a number of existing sources, including: (i) national survey data, such as the National Health and Nutrition Examination Survey [NHANES] in the United States; the National Food Survey of Great Britain; or the Living Costs and Food Survey, also in the UK); (ii) other public data such as price data from the National Institute of Statistics and Geography in Mexico; and (iii) data collected by private research companies, such as the Nielsen Homescan Panel (e.g. in the UK, US and Australia). Two modeling studies used simulated cohorts: Gortmaker and colleagues [7] used a simulated cohort representative of the 2015 US population, and Zhang and colleagues [8] developed a simulation model to represent an adult population in California (which itself drew from a national survey and other empirical research).

We acknowledge that certain context-specific factors will influence how clearly a tax is visible to the consumer, and this is likely to have an important influence on how consumers respond. In the UK and other European countries taxes on food and beverages are incorporated into the price displayed on the shelf, such that the consumer’s purchasing decision is made on the post-tax price. In North America, taxes usually appear on the sales receipt as a non-itemized addition to the bill. This is likely to result in a lower level of transparency of the gross price of an individual product, and less sensitivity to tax-related price changes. For example, an evaluation of SSB sales taxes in two US States observed that a significant reduction in SSB consumption did not occur, and the authors argue that this may be because the tax was not displayed on the shelf [9]. However, the majority of studies included in this review did not specify whether purchasers are aware of the tax at time of purchase decision.

### Thematic results of systematic review

This section is divided into sections addressing the five questions outlined in the methods.

#### How (if at all) do particular health taxes change consumption behaviours and what do we know about the health-related impacts of such taxes?

Like make taxes on tobacco and alcohol products [3, 10–12], the majority of taxes on healthy food and non-alcoholic beverages were intended to improve population health by reducing product consumption (see [13]). Definitions of ‘unhealthy’ or ‘junk’ foods vary within included studies but were commonly defined to target foods high in fat, salt and/or sugar [14]. In some cases, definitions included products high in caffeine or products that had been subjected to intensive processing, such as processed meat [15]. For non-alcoholic beverages, the most common targets of taxes were SSBs, which can include soft drinks or soda, cordials, other sugar-added juices, and ‘isotonics’ [16–18]. A small number of studies also included milk-based products (e.g. milk desserts [19]) or full fat or high-sugar milk [20, 21].

Taking a reduction in product consumption as the primary aim of these taxes, Table 1 summarizes the number of studies, by study design type, which found either positive health impacts or no/negative health impacts. Two modeling studies [16, 18] have been included in counts of ‘positive’ and ‘negative’ impacts because they found both positive and negligible/negative health impacts. One mixed methods study using modeling and evaluation methods was also double-counted in Table 1 as it found both positive and negative health impacts [17].

**Table 1:** Number of studies identifying positive health impacts by study design type

Study design	Number of Studies Included	Number of studies that found a positive health impact	Number of studies that found no, or negative, health impacts
Modeling	17	16	3
Experimental	0	0	0
Evaluation	8	4	4
Mixed method^a^	1	1	1
Total	26	21	9

^a^Both modeling and evaluation

Table 1 suggests that modelling studies (e.g. [18–20]) were more likely to find a positive health impact than evaluations [24–26], perhaps because these studies often model the impact of higher tax rates than those that have been evaluated.

Nonetheless, four evaluation studies identified positive health impacts of the (generally lower level) taxes they assessed. Evaluating the effect of the Danish fat tax (2011-2013) on risk of ischemic heart disease (IHD), Bodker and colleagues found marginal changes in population risk of IHD [24]. Smed et al., also evaluating the Danish tax, used retail scanner data to estimate the impact of the tax on population risk of IHD, stroke and heart failures [26]. Although the results for each disease varied, the study estimated there was a small overall reduction in mortality from non-communicable diseases (mostly in men and young women). Overall, the researchers estimated the tax averted or delayed 123 deaths per year, although given the absence of a control group, a causal link to the tax cannot be drawn [26]. In another context, Fletcher and colleagues evaluated the impact of changes in soft drink taxes at state level (which were, on average, around 3%) in the United States on BMI, obesity, and overweight [25]. Using nationally-representative data, the authors found that soft drink taxes had a statistically significant, albeit small effect (decrease) on BMI, obesity, and overweight. These three studies caution that low taxes on unhealthy products may influence consumption behaviour, however are unlikely to lead to substantial population health changes. In another American-focused study (although using a different national dataset from Fletcher et al.), Kim and Kawachi found that between 1991 and 1998, states without taxes on SSBs or snack foods, or states that had repealed a similar tax, were greater than four and 13 times as likely, respectively, than states with a tax to experience a relatively high increase in population obesity [28].

The four included evaluation studies that found no, or negative, health impacts were conducted in the United States context and examined the effect of SSB taxes and weight-related measures (e.g. BMI or obesity) in young people. In contrast to their study above, which examined adult populations, Fletcher and colleagues found that current state SSB taxes in the United States had no significant effect on children’s weight, finding that in fact children consumed more calories from SSBs in states that had implemented an SSB tax than in states that had not (although this was not statistically significant) [29]. The researchers posit that in this case, the consumers are likely not reacting to the small and possibly hidden taxes on SSBs. In a separate article [30], Fletcher et al. again found existing SSB taxes did not significantly reduce weight in young people, which was attributed to youth substituting other high-calorie drinks such as whole milk. Using cross-sectional data on American adolescents, Powell and colleagues found no statistically significant associations between BMI and state-level SSB taxes in grocery stores and vending machines [31]. Sturm et al. also examined existing SSB taxes in the United States and their impact on young people’s obesity. Using longitudinal data from an early childhood study, the authors found no significant relationship between current taxes (usually no higher than 4% in grocery stores) and children’s SSB consumption or obesity [32]. In contrast to modelling studies which often model taxes at higher rates (and more often find positive health impacts), the above evaluation studies provide valuable insight into the effectiveness of existing taxes implemented at lower rates.

Table 2 summarizes the number of studies, by tax rate, distinguishing between rates of less than 20% and those of 20% or more (since this is the most commonly used threshold across the literature reviewed (e.g. [21, 29, 30])) and product type, distinguishing between SSBs and food products. In total, 22 studies are included in Table 2. Again, certain studies are included more than once if they considered separately taxes of different rates or the health effects of tax rates on different products [17, 20, 35]. Studies which did not make the tax rate explicit or which focused on taxes applied to both SSBs and food are excluded from the table. Studies involving taxes applied to sugar/sweeteners are classified as food product taxes.

**Table 2:** Number of studies identifying health impacts by tax rate and product

Tax rate and product	Number of studies that found a positive health impact	Number of studies that found no, or negative, health impacts
Tax rate of <20% SSBs	3	5
Tax rate of 20% + SSBs	8	0
Tax rate of <20% food products	4	3
Tax rate of 20% + food products	3	0
Total	18	8

Taken collectively, the studies in Tables 1 and 2 suggest there is considerable evidence that taxes on SSBs and unhealthy food products can have positive health impacts. However, as Table 1 demonstrates, the majority of studies included in this review were based on modelling or experiments involving *potential* taxes. This is despite the fact that instances of such taxes exist in many countries. For example, Finland, France, Latvia, and Hungary have implemented taxes on both foods and beverages high in added sugar [36]; Portugal and Hungary have implemented taxes on products high in salt [36], Hungary has implemented a tax on foods high in fat, and Denmark introduced (and later repealed) a tax on saturated fat [36]. In addition, there have been several instances of taxes on sugar-sweetened beverages, including in Mexico, two US cities and various small island states [37–39]. This suggests there are substantial opportunities for developing the available research evidence concerning the evaluation of the health impacts of taxes that have been implemented on food and beverages.

Table 2 shows that evidence in support of applying taxes to unhealthy products is strongest for taxes on SSBs set at a rate of 20% or more of the price (e.g. see [13]). The evidence for health impact from lower taxes on SSBs is weaker, with the number of studies finding positive health impacts equal to those that found no positive impact. The evidence of taxes on food products is more mixed and difficult to assess since many of the studies involve complicated bundles of taxes (e.g. [33]).

Of the small number of studies that commented on the relationship between the type of tax applied and health impacts, there was a consistent finding that specific taxes (i.e. a fixed value based on the quantity, size or weight of the product) are associated with stronger health benefits than *ad valorem* taxes, which are proportional to the price. Applying specific taxes means that all products covered by the tax are taxed equally. In contrast, *ad valorem* taxes mean that more expensive, premium products attract a higher tax, which tends to increase price differences across brands, providing more scope for consumers to respond to new or higher taxes by selecting a cheaper brand or version (e.g. [37]). This is a finding which parallels evidence regarding tobacco taxes [2].

Looking in more detail at the studies that involved evaluating taxes that had been implemented (rather than those modelling the effects of potential taxes), most of which focused on the US, the evidence for the impact of taxes on consumption patterns and health outcomes is mixed. As of 2014, most US states had applied some level of taxation to soft drinks, largely for revenue raising purposes [40], and these do not appear to have had a significant impact on consumption of soft drinks. For example, in an analysis of sales data from Maine and Ohio, one study found that the rate of taxation on the price of soft drinks was, at 5.5% and 5% respectively, insufficient to create a statistically significant change in consumption [9]. This finding was consistent with an evaluation by Sturm and colleagues [32], which found that existing taxes on soda, at rates that are typically around 4% in grocery stores in most states, did not have a statistically significantly effect on soda consumption and obesity rates in the US [32]. Other countries have implemented a higher rate of tax on soft drinks than in the US. For example, in September 2013, Mexico implemented a 10% tax on soft drinks and an 8% tax on unhealthy snacks [41]. It is estimated that the tax on soft drinks contributed to a 6% average decrease in purchasing of taxed beverages by December 2014, with purchasing reductions being greatest in low income households [42].

Twenty-three studies considered the estimated or actual health impacts of taxes applied in conjunction, or comparison, with a range of other health-related interventions. Several studies examined the impact of using subsidies – i.e. negative taxes on ‘healthy’ products - alongside taxes on ‘unhealthy’ products. Most often, the subsidies were applied to fruit and vegetables [18, 21, 43]. Other ‘healthy’ products that were included in the analyses observed were grain-based products high in fibre, fresh fish, and bottled water [14, 44, 45].

Several of these studies indicate that a combination of taxes and subsidies can have large behavioural and health impacts [44, 46]. However, it is difficult to ascertain from the findings reported in these modelling studies and experiments whether taxes, subsidies or a combination of the two, are most effective in achieving such impacts. Many of the studies point out, however, that a key advantage of employing subsidies in combination with taxes is that the former can help to offset the inequitable (or regressive) burden of the latter.

A number of the studies considered (likely or actual) differential health impacts by population group. Of these, eight found that taxes on food/beverages were likely to have a greater impact on younger population groups [22, 23, 26, 27, 47–50] and 15 found that public health impacts are likely to be largest for lower income groups [22, 25, 27, 32, 33, 42, 44, 46, 48, 49, 51–56]. In contrast, two studies [23, 34] found no significant differences between income groups. This suggests that taxes on unhealthy food and beverages may contribute to addressing health inequalities, but that more research is required. As we discuss in more detail later on, 27 of the included studies highlighted the regressive burden of taxes on food and beverage products, suggesting that there is a balance to be struck between the inequitable burden of ill-health and the inequitable burden of taxes.

Overall, there is considerable evidence that high tax rates (i.e. those that raise the unit price by 20% or more) on beverages are likely to have a positive impact on health behaviours and outcomes. The evidence is similar for taxes targeting unhealthy foods, though there are a smaller number of studies and the taxes in question were often more complicated. This finding is consistent with a recent review, which found that food taxes and subsidies are associated with changes in consumption behaviours [57] and also reflects what is known about alcohol and tobacco taxes [3, 10–12]. However, as noted, it is apparent that such tax rates are far higher than those that have actually been implemented. Hence, it may be that, as Fletcher and colleagues noted, “typically imposed beverage taxes aren’t large enough or transparent enough to lead to meaningful behaviour change.” ([23], p.1064).

#### Can health taxes on manufacturers and retailers change behaviours?

Most studies focus on health taxes that are applied at the point of sale, and are intended to try to motivate consumers to change their consumption decisions. It should be noted, however, that a tax on manufacturers may or may not be intended to change behaviour in relation to a finished good, but rather to the use of specific raw materials (ingredients).

We identified three studies targeted at manufacturers or retailers. One such study, by Miao, Beghin, & Jensen [19], modelled an approach to taxation that targeted the process of adding sweeteners to products, and compared this with a consumption tax on sweetened products [19]. The rationale was that a tax on sweetener would incentivize producers of high-sugar products to reduce sweeteners in food processing by increasing the unit cost of these products to the manufacturer (while the consumption tax would change consumer-purchasing patterns). As the tax increases the cost of production, suppliers (manufacturers) may respond by increasing the price of the finished good and/or decreasing supply of the product in response to the reduction in profits they make by selling it. However, in some cases, it may not be economically advantageous for suppliers to pass on higher costs to consumers, or to reduce supply in response to higher costs. In this instance, it may be regarded as beneficial to change the formulation of product, e.g. by reducing the fat or sugar content. In this case, the authors conclude that both approaches are potentially effective, but that taxing added sweeteners is likely to have a smaller impact on consumers’ real expenditures than taxing final products.

Another study, which assessed the impacts of a set of complex unhealthy food taxes implemented in Hungary, undertaken by Hungary’s National Institute for Health Development (cited in [56]), found that substantial changes were subsequently made to the manufacturing of certain products. A survey of manufacturers suggested that the taxing of products exceeding a minimum threshold of certain ingredients such as sugar and fat led 40% of manufacturers to modify their recipe; 30% removed the ingredient entirely, and 70% reduced the level of the ingredient [58].

In theory, Scotland’s public health supplement on large retailers selling tobacco and alcohol had the potential to discourage retailers from selling either alcohol or tobacco (the latter was a more likely outcome, given the relative profitability of the two types of products). In practice, however, this evaluation found that the level of the tax was too low to stimulate changes in retail practice, which enhanced the predictability of the associated revenue (as discussed above) [4].

#### Do taxes that target health-damaging products succeed in providing additional fiscal revenues?

Most studies find that, while taxes on products reduces demand for those products, they add to fiscal revenues (e.g. [57]). However, our review suggests that the associated revenue streams may be subject to a significant degree of volatility. As human responses to price changes are complex, and vary by context and over time, the extent of the revenues likely to be raised by health taxes is difficult to estimate with precision. Such estimates are particularly vulnerable to uncertainty over longer periods. For example, Zhen and colleagues [56] examine the interaction of taxes on SSBs with human habit formation, in which decreases in consumer purchasing attributable to SSB taxes are larger in the long-term as habits are gradually broken, resulting in progressively lower tax revenues. However, the authors acknowledge that revenues could also increase over time as consumers became more accustomed to higher prices (e.g. [52]).

It seems clearer that the revenues generated by consumer taxes are easier to predict, and are likely to be higher, when specific, rather than *ad valorem*, taxes are employed. For a more detailed explanation of this point, see [2] in relation to tobacco taxation.

We identified one study that assessed the impacts of a tax applied to retailers of alcohol and tobacco which, being set on the basis of the value of premises, was difficult for retailers to avoid or pass on to consumers. Hence, unusually for a tax framed as health-related, the entities from whom the taxes were collected bore the full burden of the tax. The ‘public health supplement’ was a levy on large retailers of alcohol and tobacco products implemented in Scotland 2012-2015. The study found that the revenue from this type of tax (administered through a supplement to the business rates system) was highly predictable over a three-year period [4]. Indeed, although the tax was relatively short-lived (it was discontinued after 3 years in the face of resistance from large retailers) the revenue raised in this period was slightly above the government’s predictions. The case study shows that taxes can be designed in such a way as to enhance the predictability of the associated revenues. However, by making the tax uneconomic for retailers to try to avoid (i.e. by changing their policies with respect to selling alcohol or tobacco), while largely insulating consumers from the burden of the tax, there was no mechanism for stimulating desirable changes in the supply and consumption of such products, or reducing associated health harms [4].

#### What is the degree of support among public and policy communities for non-traditional health taxes and are there means of increasing support?

Several papers provided insights into three broad categories of factors affecting the feasibility and implementation of new health taxes. The first concerns public opinion regarding proposed, or actual, taxes. Here, studies consistently find that public support for new consumption taxes, or tax increases, is low [60–63], though some suggest that there is public or ministerial support for sugared beverage taxes in some contexts [39, 64, 65]. A four-country study in the Western Pacific region by Thow et al. suggests that, although governments are ultimately concerned with raising revenue, framing a tax around health promotion can assist in getting such a tax onto the policy agenda in the first place [39]. For example, a tax on unhealthy food products introduced in French Polynesia in 2002 was framed as a response to concerns regarding poor nutrition and non-communicable disease [39]. The tax enjoyed broad ministerial support, which was attributed to the tax’s earmarking for public health and other cultural, educational, and youth-focused initiatives, which benefited seven of the 17 ministers in the government [39]. More generally, support among the general public seems to be higher when credible commitments are made to earmarking funds for specific health activities and objectives, such as subsidizing healthier foods or targeting child obesity (e.g. [63, 64]).

We identified a smaller number of studies that considered the media coverage of proposed or actual health taxes which might be expected to both reflect and shape public opinion. In some cases, such as the Danish fat tax and the Scottish public health supplement (described above), industry interests opposed to the tax have been able to dominate media coverage, helping to secure further opposition to the tax (which, in both these cases, was eventually dropped) [4, 24, 66]. In contrast, [67] analysis of an SSB tax in Mexico provides an example of a supportive media, in which public health advocates successfully utilized media campaigns to raise the public and political profile of the issue and communicate with the public. Less positively, [68] analysis of debates about potential soda taxes in three US states found that, despite public health advocates’ ability to dominate media coverage with pro-tax messages, none of the proposals were implemented. Hence, while media support for a health tax proposal may be important for it to succeed, it is not sufficient [38].

Twelve studies considered policy design and implementation factors shaping the fate of proposed and actual health taxes. Studies considering political factors suggested that political support for, and opposition to, health taxes are likely to be key to understanding why some taxes are implemented and others are not (or why some taxes are repealed) (e.g. [3, 20, 31, 64, 65, 67, 68]). These studies also suggest that opposition to health taxes can develop relatively quickly. For example, [69] highlights how political opposition to a proposed soft drink tax arose in 2009 in New York State and contributed to the tax proposal being withdrawn prior to implementation, while in contrast [67] outlines the substantive advocacy efforts in Mexico to combat multi-stakeholder opposition, leading to Mexico’s tax being successfully implemented.

In a study of taxes implemented in Pacific Island nations, [39] identify industry lobbying in Fiji as a cause of the decision to abolish the country’s domestic excise tax on SSBs. Two studies of the short-lived Danish fat tax both argued that lobbying by food industry interests helped secure political opposition to the tax once it had been implemented, while there appears to have been only limited efforts by the government to secure broader public support. In the absence of such support, political opposition increased and a decision was taken to drop the tax after less than a year (in advance of any analysis of its health impacts) [24, 66]. The assessment of Scotland’s levy on large retailers reached similar conclusions in relation to industry opposition and the political sustainability of a policy framed as a health tax for which the health rationale appeared to shift over time [4].

#### What are the key critiques of health taxes and their implementation and what options exist to manage these challenges?

We identified three key criticisms of taxes on unhealthy products. Twenty-seven of the included studies highlighted the regressive nature of the health tax examined (e.g. [58, 70, 71]). Poorer groups may be more price sensitive than other groups, and therefore more likely to change their behaviour in response to a tax. In this sense, taxes may play a role in addressing health inequity. In addition, it is important to acknowledge that the regressivity of existing taxes does not necessarily imply that tax *increases* will be regressive since, if poorer consumers are more responsive the burden of the tax may shift more to wealthier consumers [74]. This argument is often made in relation to tobacco taxation – see [2]. However, if price elasticity is low (as is typical for many unhealthy products), those with lower incomes who continue to buy these products have less to spend on basic needs, such as housing, heating, and healthy food, potentially at the expense of their health and general welfare. Available research does not sufficiently address the question of whether, among low-income consumers, the overall benefits of tax-induced price increases (i.e. reducing consumption of unhealthy products) outweigh the risk of harm from financial hardship for those who do not reduce consumption. More generally, existing evidence concerning outcomes in terms of progressivity/regressivity is limited by the fact that nearly all studies addressing this issue that we identified were based on modelling or predictive experiments. If this particular combination of fiscal measures has occurred in practice, we were unable to find any evaluation studies that covered the issue of regressivity.

For policy actors concerned about the regressive potential of taxes on unhealthy products, one potential response to this would be, as noted above, to use the revenue from such taxes to subsidize other ‘healthy’ foods, such as fruit and vegetables. In this way, it may be possible to put together a package of policies in which there can be some confidence that the overall impact on poverty will be negligible [72].

A second criticism, put forward by Fletcher et al., is that food and beverage taxes may simply lead to consumers substituting the taxed products for similar, non-taxed alternatives which are not necessarily healthier, such as sports drinks or juice [29]. This is an issue that has also been identified in the context of differential taxes on different types of tobacco products [2]. It implies that there is a need to carefully assess behavioural changes in response to taxes intended to achieve health goals, and that policymakers need to stay alert to the possibility that such taxes may need to be revised or expanded in response to changing behaviours.

The third key criticism is that implemented taxes are often too low to have a meaningful health impact, a criticism that is borne out by the empirical evidence, as noted above [29]. It may be appropriate to consider the level of a tax before deciding whether or not it is appropriate to frame it as a ‘health tax’. Lower and incremental taxes are more likely to provide a stable source of revenues (which may, or may not, be spent on health-related activities) but they are less likely to achieve behaviour changes [59, 71].

In addition to these three criticisms, it is evident that, while framing new taxes or tax increases as mechanisms for increasing health spending may increase public support, funds may not always be clearly earmarked in practice [2, 4]. Where this occurs, this may undermine support for such taxes in the longer-term.

Looking back across our five research questions, it is apparent that the results of our review are consistent with those focusing on traditional excise taxes on alcohol and tobacco. Those reviews show that increasing taxes leads to reduced consumption among the population and can be a valuable source of revenue for government [10, 73–75]. A review by Chaloupka and colleagues shows that revenue from tobacco taxes may be more reliable than those discussed in the present review, however, because there are fewer substitutes available for tobacco products, and the demand for them is therefore relatively inelastic [74]. With regard to public support for alcohol and tobacco taxes, studies find greater public support for these approaches when the tax is earmarked for healthcare or for combating tobacco- or alcohol-related harms [74, 76]. The concern with the regressivity of health taxes is also relevant for alcohol and tobacco. A recent study confirmed that alcohol taxes are regressive, although the authors interpreted this effect to be small [77]. A review by Hill and colleagues found that tobacco price increases via taxes has a greater impact on low-income groups compared with those with high incomes (although, similar to the present review, this effect is argued to be positive given its potential to reduce socioeconomic inequalities) [78].

## Discussion

Although extensive efforts have already been made to understand the impacts of, and responses to, tobacco and alcohol excise taxes [2, 3, 10, 12], this review is the first attempt to systematically identify and synthesize this broader literature on health taxes. In this section, we focus on summarizing the key implications of the review for future research and policymaking.

The review highlights that there has been a rapid increase in research in this area, most of which focuses on taxes on food products or nutrients (indeed, in the time between updating our searches and submitting this paper, several further studies have been published on this topic, (e.g. [72, 74])). Of the studies included in this review, the majority (*n* = 93) focus on health taxes in high income settings (particularly the USA, *n* = 50). However, the findings are likely to be highly relevant for policymakers in developing country contexts, in which efforts to provide universal health coverage require the effective utilization and expansion of domestic public sector financial resources [5].

Nearly half of developing countries have tax shares of less than 15% of GDP [79], and many are already operating near their tax capacity – suggesting that improvements in tax collection alone will not provide adequate resources for health. Indirect taxes levied on health-damaging goods offer a potentially attractive source of additional fiscal space as, in addition to raising revenue, they are a proven method of influencing individual behaviour, reducing negative externalities on others, and curbing the incidence of the costly NCDs caused by consumption of such goods. Taxes on SSBs recently passed in California and the UK provide potential for additional evaluative case studies. Methodologically, the review identified a strong preference for predictive research (especially modelling) over evaluation. The review found that modelling studies tend to predict more positive health impacts than evaluations (likely explained by the fact the taxes researchers have modelled have generally been higher than those that those actually implemented), suggesting that more evaluative research is needed as policymaking in this area evolves.

Turning to policy, our findings suggest that a number of taxation tools are available to policymakers - and that each has advantages and disadvantages. The choice of taxation tool to apply will depend on the overall aim of the tax and the context in which policymakers seek to implement it. Overall, we identify four substantive results. First, while there appears to be a large number of innovative health taxes being implemented, most involve expanding the number of unhealthy commodity products (notably sugar) that are taxed. This is a possible source of concern since public support for new commodity taxes tends to be low, and high public or political support is likely to be required for taxes to be initiated and sustained. Furthermore, as examples such as the short-lived Danish fat tax, the Fijian SSB tax [24, 39] and the (unsuccessful) attempts of several US States to introduce SSBs taxes [69] illustrate, such policies are likely to be challenged by strong industry interests. In the case of Scotland’s Public Health Supplement, there was no strong public opposition to the tax (which was not easily passed on to consumers of targeted products), but the government faced extremely strong opposition from affected businesses and, in that context, opted to discontinue the tax after 3 years [4].

Second, our findings suggest that commitments to earmarking the revenue from health taxes for specific purposes, such as funding health system improvement or obesity prevention, can increase public and political support for taxes [39, 60, 80, 81]. Earmarking revenue for health spending is one means of encouraging support from the public health community (e.g. NGOs, researchers and practitioners) which may help offset the influence of industry interests. However, as both the experience of the Public Health Supplement [4] and earlier tobacco tax policies have shown [12], governments may fail to abide by initial earmarking commitments once taxes have been implemented, and this provides an obvious lobbying focus for those opposed to the tax, undermining public and political support for its existence [12].

Third, there are potential mechanisms for reducing the regressive nature of health taxes on consumer products. Options identified in this review were: (i) using the revenue raised from taxes to subsidize healthier products; and (ii) targeting ingredients used in the production of certain products, instead of the product itself (as seen in the UK sugar tax). In the latter case, producers are incentivized to remove or decrease the targeted ingredient from the product. Assuming that any related manufacturing costs are not passed on to the consumer, it is plausible that the health impact goals may be attained without negatively affecting those on lower incomes.

Fourth, our results show the importance of clear prioritization of objectives when designing taxes. Some objectives may be in conflict. For example, our results (and the previous reviews of tobacco and alcohol product taxes) show that, if the purpose of a tax is to achieve health gains via behavioural change, it must be set at a sufficiently high level. For SSBs, taxes of 20% or more of the sale price are most likely to be effective in this respect, whereas the evidence is much less clear regarding lower level taxes (such as many of those levied on SSBs to date). In contrast, if the aim of a new tax is to raise revenue (whether these are earmarked for health purposes or not), then taxes set at a rate that is high enough to incentivize behavioural changes may be less desirable, since this will reduce the stability of associated revenues, and a lower rate may be more appropriate.

A number of limitations to this study exist. The size of the review necessitates that not all titles and abstracts could be screened by all authors. In addition, both the variable methodological approaches of included studies and our commitment to providing an inclusive overview of existing evidence meant it was impossible to apply a uniform method of critical appraisal across studies. Thus, it is possible that the ‘weight’ attached to low quality studies is similar to that of high-quality studies. We are also limited by the evidence available, and the relative lack of evaluation studies in particular.

We have, however, brought together studies from multiple disciplines, including public health, nutrition, health policy, economics, medicine, and psychology, allowing us to provide a comprehensive overview of the policy lessons regarding health taxes. This is, to our knowledge, the first attempt to provide a broad overview of the evidence relating to these taxes. It therefore addresses a series of questions that policy actors considering health taxes (or tax increases) ought to consider in designing any new measure and identifies important gaps for future research to address.

## Conclusions

If the primary policy goal of a health tax is to reduce consumption of unhealthy products, then current evidence supports the implementation of taxes that increase the price of products by 20% or more. However, where taxes are effective in changing health behaviours, the predictability of the revenue stream is reduced. Hence, policy actors need to be clear about the primary goal of any health tax and frame the tax accordingly – not doing so leaves taxes vulnerable to hostile lobbying. Conversely, earmarking health taxes for health spending tends to increase public support so long as policymakers follow through on specified spending commitments. With more and more countries implementing new kinds of health taxes, there are numerous opportunities for real-world evaluations to substantially strengthen the current evidence-base.

## Additional file

Additional file 1: Complete Search Strategies. The additional file provides all search strategies used for the peer-reviewed and grey literature databases searched for this review. (DOCX 105 kb)

## Abbreviations

BMI: Body mass index
IHD: Ischemic heart disease
NGOs: Non-governmental organisations
NHANES: National Health and Nutrition Examination Survey
SSBs: Sugar-sweetened beverages
UK: United Kingdom
US: United States
