# Supplementary material for: Policy lessons from health taxes: a systematic review of empirical studies
Source: BMC Public Health. 2017 Jun 19;17:583. doi: 10.1186/s12889-017-4497-z (PMC5477308; doi:10.1186/s12889-017-4497-z)
Supplement: Additional file 1: — Complete Search Strategies. The additional file provides all search strategies used for the peer-reviewed and grey literature databases searched for this review. (DOCX 105 kb) [file 12889_2017_4497_MOESM1_ESM.docx]

**PubMed**

Search string:

(((health) AND (tobacco OR cigar* OR alcohol OR drink* OR beer OR wine OR spirits OR made-wine OR cider OR perry OR food OR soda OR beverage* OR sugar OR fat OR "sin tax")) AND (tax*[Title/Abstract] OR levy[Title/Abstract] OR levied[Title/Abstract] OR excis*[Title/Abstract])) NOT ("taxonomy" OR "syntax" OR "excision" OR "taxonomic" OR "taxonomically" OR "taxane" OR "taxi" OR "taxonic" OR parasit* OR microbial OR phenotyp*)

Filters: 1990-2015; Humans; English

Note: Second search completed 2^nd^ May 2016; Date: 2015-2016; English.

**OVID: Embase, Global Health, PsychINFO**

Search String:

((health and (tobacco or cigar* or alcohol or drink* or beer or wine or spirits or made-wine or cider or perry or food or soda or beverage* or sugar or fat or "sin tax")).mp. and (tax* or levy or levied or excis*).ab.) not ("taxonomy" or "syntax" or "excision" or "taxonomic" or "taxonomically" or "taxane" or "taxi" or "taxonic" or parasit* or microbial or phenotyp*).mp. [mp=ti, ab, hw, tn, ot, dm, mf, dv, kw, bt, id, cc, tc, tm]

Filters: English; Human; 1990-2015; Journal Article

Note: Second search completed 2^nd^ May 2016; Date: 2015-2016; English

**Web of Science**

Search String:

TOPIC: (health) AND TOPIC: (tobacco OR cigar* OR alcohol OR drink* OR beer OR wine OR spirits OR made-wine OR cider OR perry OR food OR soda OR beverage* OR sugar OR fat OR "sin tax") AND TOPIC: (tax* OR levy OR levied OR excis*) NOT TOPIC: ("taxonomy" OR "syntax" OR "excision" OR "taxonomic" OR "taxonomically" OR "taxane" OR "taxi" OR "taxonic" OR parasit* OR microbial OR phenotyp*) ANDLANGUAGE: (English)

Filters: 1990-2015

Note: Second search completed 2^nd^ May 2016. Date: 2015-2016

**EBSCOhost: Academic Search Complete, Business Source Complete, SocINDEX with Full Text, EconLit, Medline**

Search String:
health AND ( tobacco OR cigar* OR alcohol OR drink* OR beer OR wine OR spirits OR made-wine OR cider OR perry OR food OR soda OR beverage* OR sugar OR fat OR "sin tax" ) AND AB ( ( tax* OR levy OR levied OR excis* ) NOT ( "taxonomy" OR "syntax" OR "excision" OR "taxonomic" OR "taxonomically" OR "taxane" OR "taxi" OR "taxonic" OR parasit* OR microbial OR phenotyp* )

**Limiters** - Scholarly (Peer Reviewed) Journals; Published Date: 19900101-20151231; Language: English; Publication Type: Academic Journal; Language: English; English Language; Human; Language: English

Filters: 1990-2015; Scholarly (Peer Reviewed) Journals; English

Note: Second search completed 2^nd^ May 2016. Date: 2015-2016; English language; didn’t filter out for ‘Humans’

**Scopus**

Search String:

( ALL ( health )  AND  ALL ( tobacco  OR  cigar*  OR  alcohol  OR  drink*  OR  beer  OR  wine  OR  spirits  OR  made-wine  OR  cider  OR  perry  OR  food  OR  soda  OR beverage*  OR  sugar  OR  fat  OR  "sin tax" )  AND  TITLE-ABS-KEY ( tax*  OR  levy  OR  levied  OR  excis* )  AND NOT  ALL ( "taxonomy"  OR  "syntax"  OR  "excision" OR  "taxonomic"  OR  "taxonomically"  OR  "taxane"  OR  "taxi"  OR  "taxonic"  OR  parasit*  OR  microbial  OR  phenotyp* ) )  AND  SUBJAREA ( mult  OR  medi  OR nurs  OR  vete  OR  dent  OR  heal  OR  mult  OR  arts  OR  busi  OR  deci  OR  econ  OR  psyc  OR  soci )  AND  PUBYEAR  >  1989  AND  ( LIMIT-TO ( DOCTYPE ,  "ar" ) ) AND  ( LIMIT-TO ( SUBJAREA ,  "SOCI" )  OR  LIMIT-TO ( SUBJAREA ,  "ECON" )  OR  LIMIT-TO ( SUBJAREA ,  "BUSI" )  OR  LIMIT-TO ( SUBJAREA ,  "ENVI" )  OR  LIMIT-TO ( SUBJAREA ,  "MULT" ) )  AND  ( LIMIT-TO ( LANGUAGE ,  "English" ) )

Filters: 1990-2015; Articles; English

Limited to subject area: Social Sciences, Economics, Business, Environment, Multidisciplinary

**ProQuest (IBSS Online and ASSIA)**

Search String:

health AND (tobacco OR cigar* OR alcohol OR drink* OR beer OR wine OR spirits OR made-wine OR cider OR perry OR food OR soda OR beverage* OR sugar OR fat OR "sin tax") AND ab(tax* OR levy OR levied OR excis*) NOT ("taxonomy" OR "syntax" OR "excision" OR "taxonomic" OR "taxonomically" OR "taxane" OR "taxi" OR "taxonic" OR parasit* OR microbial OR phenotyp*)

Filters: 1990-2015; Peer-reviewed; Scholarly Articles, English

Note: Second search done 2^nd^ May, same search string as above, Date: 2015-2016
